# Comment on “Long-term Outcomes of Parenchyma-sparing and Oncologic Resections in Patients With Nonfunctional Pancreatic Neuroendocrine Tumors <3 cm in a Large Multicenter Cohort”

**DOI:** 10.1097/AS9.0000000000000266

**Published:** 2023-03-14

**Authors:** Yufan Meng, Zhiyao Fan, Jian Yang, Yongzheng Li, Hanxiang Zhan

**Affiliations:** From the Division of Pancreatic Surgery, Department of General Surgery, Qilu Hospital, Shandong University, Jinan, Shandong Province, China.

Due to the dearth of prospective randomized clinical studies and lack of high level evidence, surgical decision-making in small nonfunctional pancreatic neuroendocrine tumors (pNET) has been controversial. We read with interest the recently published article by Dr. Ferrone et al.^1^ This study found that parenchyma-sparing resections (PSR) and lymph node-sparing resections do not affect patients’ long-term survival; they also have advantages in reducing blood loss and shorting operation time. We extend our congratulation to this excellent study with holds several advantages, including four high-volume pancreatic surgery centers, a large sample size of 810 patients, comprehensive postoperative complication data, and long follow-up period up to 208 months. However, we would like to raise some detailed issues about this study and share our opinions.

First, parts of the data published in the research puzzled us. As shown in Table 1, 111 of 221 patients who received PSR underwent lymph node (LN) dissection, and the mean number of positive LNs identified was 0.9 in 1.4 LNs harvested. Although the surgeon’s selective dissection is an important influence factor, this is a particularly high percentage indeed for enucleations and central pancreatectomies. Furthermore, 15 patients receiving PSR were finally identified with positive nodal status according to the data provided in Table 1. By analyzing of calculations, a total of 100 positive LNs were removed and the mean number of positive LNs was 6.7 among PSR patients who were positive nodal status. If multiple suspicious enlarged lymph were observed in the surgery, continuing PSR surgery is probably not the optimum strategy. At the heart of the debate, is whether patients can benefit from LN dissection or whether LN metastasis affects prognosis and survival outcome. Previous studies have concluded that LN metastasis rates were 15.8% and 11.5% in G1 and <2 cm pNET, respectively.^2^ LN metastasis conspicuously decreases survival outcomes. These results are in agreement with the main conclusions presented in this work. A national cancer database retrospective study including 2664 patients indicates that dissected lymph cannot bring additional treatment benefits to pNET patients undergoing surgery.^3^ Therefore, it is hard to reach a uniform conclusion. European Neuroendocrine Tumor Society recommends the systematic removal of LNs in the peritumoral area for all pNET operations.^4^ However, American Hepato-Pancrcato Biliary Association advocated removing suspicious LNs.^5^ One other matter of debate is the extent and number of LN dissections is difficult to pinpoint the determinants. As the authors showed in this paper, tumor location, tumor size, and surgical methods significantly affect the number of LNs harvested. Although the positive LNs are more likely to be identified if more LNs are examined, it may increase the operation time, blood loss, and postoperative complications. Maithel et al reported that 5-year recurrence-free survival increased by more than 20% for accurate staging when distal pancreatectomy removes 7 or more LNs.^6^ Adequate preoperative imaging evaluation plays a pivotal role in making operation strategy. Hence, further research is warranted to determine the role of LN dissection in patients with pNET.

Second, during specimen collection and analysis, the decision deviation of these data should be taken into account due to the long study period. The authors included all patients with nonfunctional pNET <3 cm who received surgical treatment from 2000 to 2021. The optimal therapeutic method has been changed over the years. In the latest version of the North American neuroendocrine tumor Society consensus, pancreatic tumors smaller than 1 cm in size chose to receive observation in the follow-up instead of surgery, and 1 to 2 cm in size should be individualized.^7^ And early treatment may be more radical, which recommends surgery for primary pNET without inherited pNET syndromes.^8^ Because the surgical strategy and the recommendation of treatment have significantly changed, it makes a number of nonfunctional pNET with low-grade and low-risk factors excluded from analyses. This may probably result in a higher LN metastatic potential and a poorer overall prognosis in recent years. This selection bias may not only affect the operation mode but also affect the survival outcome of patients. The authors neither distinguish among periods nor perform a standard Cox regression on tumor size and surgical methods. In addition, oncologic resection of the head and tail of the pancreas varies widely from blood loss, operative time, and postoperative complications. However, the author did not present this information in the table. Similarly, tumor location, tumor diameter, and distance from the main pancreatic duct also have great impact on surgical decision-making, we cannot get this information from the data in the study.

Finally, we notice that there exists a trend of higher postoperative complication rates for enucleations (48.4% vs 38.5%, *P* = 0.09) in Table 4. Specifically, Whipple procedure and pancreatic head enucleation were completely different in surgical steps, which demonstrate differences in numbers of aspects including operating times, blood loss, hospital length of stay and postoperative infection. Jilesen et al reported that more patients receiving enucleation of pancreatic head suffered from B or C pancreatic fistula compared with Whipple procedure (40% vs 14%, *P* < 0.001), and no statistically significant differences were presented in overall complications (*P* = 0.20).^9^ Further classification and comparison according to tumor location are suggested to be performed to obtain more precise and objective information, such as head tumor enucleation versus Whipple procedure, or distal enucleation versus distal pancreatectomy. Likewise, data from Table 2 should also be further analyzed. Alimentary tract reconstruction and removal of some organs inevitably damage psychosocial and physical functions. Therefore, we sincerely expect this topic would be intensively explored.

We are grateful for the contributions of Dr. Ferrone’s team and expect the discovery of further interesting findings in this field.

## References

[1] Bolm L Nebbia M Wei AC. Long-term Outcomes of Parenchyma-sparing and Oncologic Resections in Patients With Nonfunctional Pancreatic Neuroendocrine Tumors <3 cm in a Large Multicenter Cohort. Ann Surg. 2022;276:522–531. 35758433 10.1097/SLA.0000000000005559 PMC9388557

[2] Gao Y Gao H Wang G. A meta-analysis of Prognostic factor of Pancreatic neuroendocrine neoplasms. Sci Rep. 2018;8:7271. 29739948 10.1038/s41598-018-24072-0 PMC5940798

[3] Mao R Zhao H Li K. Outcomes of Lymph Node Dissection for Non-metastatic Pancreatic Neuroendocrine Tumors: A Propensity Score-Weighted Analysis of the National Cancer Database. Ann Surg Oncol. 2019;26:2722–2729. 31209670 10.1245/s10434-019-07506-5 PMC7436555

[4] Falconi M Eriksson B Kaltsas G. ENETS Consensus Guidelines Update for the Management of Patients with Functional Pancreatic Neuroendocrine Tumors and Non-Functional Pancreatic Neuroendocrine Tumors. Neuroendocrinology. 2016;103:153–171. 26742109 10.1159/000443171 PMC4849884

[5] Mansour JC Chavin K Morris-Stiff G. Management of asymptomatic, well-differentiated PNETs: results of the Delphi consensus process of the Americas Hepato-Pancreato-Biliary Association. HPB (Oxford). 2019;21:515–523. 30527517 10.1016/j.hpb.2018.09.020

[6] Lopez-Aguiar AG Zaidi MY Beal EW. Defining the Role of Lymphadenectomy for Pancreatic Neuroendocrine Tumors: An Eight-Institution Study of 695 Patients from the US Neuroendocrine Tumor Study Group. Ann Surg Oncol. 2019;26:2517–2524. 31004295 10.1245/s10434-019-07367-y PMC10181829

[7] Howe JR Merchant NB Conrad C. The North American Neuroendocrine Tumor Society Consensus Paper on the Surgical Management of Pancreatic Neuroendocrine Tumors. Pancreas. 2020;49:1–33. 31856076 10.1097/MPA.0000000000001454 PMC7029300

[8] Kulke MH Anthony LB Bushnell DL. NANETS treatment guidelines: well-differentiated neuroendocrine tumors of the stomach and pancreas. Pancreas. 2010;39:735–752. 20664472 10.1097/MPA.0b013e3181ebb168 PMC3100728

[9] Jilesen AP van Eijck CH Busch OR. Postoperative Outcomes of Enucleation and Standard Resections in Patients with a Pancreatic Neuroendocrine Tumor. World J Surg. 2016;40:715–728. 26608956 10.1007/s00268-015-3341-9 PMC4746212

